# Mixed methods feasibility and usability testing of a childhood obesity risk estimation tool

**DOI:** 10.1186/s12889-023-16500-2

**Published:** 2023-09-04

**Authors:** Grace Grove, Nida Ziauddeen, Paul Roderick, Ivaylo Vassilev, Jane V. Appleton, Dianna Smith, Nisreen A. Alwan

**Affiliations:** 1 School of Primary Care, Population Sciences and Medical Education, Faculty of Medicine, University of Southampton, Southampton, UK; 2 NIHR Applied Research Collaboration Wessex, Southampton, UK; 3 School of Health Sciences, Faculty of Environmental and Life Sciences, University of Southampton, Southampton, UK; 4 Formerly Professor of Primary and Community Care, Oxford Brookes University (Retired), Oxford, UK; 5 School of Geography and Environmental Science, University of Southampton, Southampton, UK; 6 NIHR Southampton Biomedical Research Centre, University of Southampton and University Hospital Southampton NHS Foundation Trust, Southampton, UK

**Keywords:** Childhood obesity, Risk prediction, Prevention, Pregnancy, Early years, Health visiting, Health inequalities, Mixed methods

## Abstract

**Background:**

A Childhood Obesity Risk Estimation tool (SLOPE CORE) has been developed based on prediction models using routinely available maternity and early childhood data to estimate risk of childhood obesity at 4–5 years. This study aims to test the feasibility, acceptability and usability of SLOPE CORE within an enhanced health visiting (EHV) service in the UK, as one context in which this tool could be utilised.

**Methods:**

A mixed methods approach was used to assess feasibility of implementing SLOPE CORE. Health Visitors (HVs) were trained to use the tool, and in the processes for recruiting parents into the study. HVs were recruited using purposive sampling and parents by convenience sampling. HVs and parents were invited to take part in interviews or focus groups to explore their experiences of the tool. HVs were asked to complete a system usability scale (SUS) questionnaire.

**Results:**

Five HVs and seven parents took part in the study. HVs found SLOPE CORE easy to use with a mean SUS of 84.4, (*n* = 4, range 70–97.5) indicating excellent usability. Five HVs and three parents took part in qualitative work. The tool was acceptable and useful for both parents and HVs. Parents expressed a desire to know their child’s risk of future obesity, provided this was accompanied by additional information, or support to modify risk. HVs appreciated the health promotion opportunity that the tool presented and felt that it facilitated difficult conversations around weight, by providing ‘clinical evidence’ for risk, and placing the focus of the conversation onto the tool result, rather than their professional judgement. The main potential barriers to use of the tool included the need for internet access, and concerns around time needed to have a sensitive discussion around a conceptually difficult topic (risk).

**Conclusions:**

SLOPE CORE could potentially be useful in clinical practice. It may support targeting limited resources towards families most at risk of childhood obesity. Further research is needed to explore how the tool might be efficiently incorporated into practice, and to evaluate the impact of the tool, and any subsequent interventions, on preventing childhood obesity.

**Supplementary Information:**

The online version contains supplementary material available at 10.1186/s12889-023-16500-2.

## Background

Childhood obesity is a significant issue worldwide, with 39 million children under 5 years classed as overweight or obese in 2020 [1]. In England, over a fifth (22.3%) of children aged 4–5 years and over a third (37.8%) of children aged 10–11 years, were classed as overweight or obese in 2021/22 [2]. Children who are obese are at five times higher risk of remaining obese into adulthood [3], and thus obesity is a driver of health inequalities over the lifecourse. Poverty [4], social inequality [5] and poor diet quality [5–7] are all linked with childhood obesity in the UK. The number of children living in relative child poverty (defined as *‘the proportion of children living in households with incomes below 60% of the median in the same year’ p.33 *[8]) in the UK has increased from 27% of children in 2013–2014, to 31% in 2019–2020 [8].

Successfully losing weight can be difficult, and challenging to sustain in the longer term [9], thus maintaining a healthy weight is preferable. Altering risk perception has the potential to influence behaviour [10]. In 2016, a WHO report *‘Ending Childhood Obesity’* highlighted the importance of a life course approach, including intervening in the preconception, pregnancy and early years periods [11]. This report focusses on six key areas*, ‘promote intake of healthy foods, promote physical activity, preconception and pregnancy care, early childhood diet and physical activity, health, nutrition and physical activity for school-age children and weight management*’ p. VII [11]. A recent systematic review [12] of 30 studies found that interventions to prevent obesity in pre-school aged children (birth to five years) were more effective when begun earlier in life, and should be prioritising families living in areas characterised by higher relative deprivation, who may be at higher risk of obesity and may find it more difficult to engage in interventions[12]. A Cochrane review also highlighted the importance of early intervention and a preventative approach, and found evidence that combined nutritional and physical activity interventions were effective in reducing risk of obesity in 0–5 year olds [13].

The pregnancy and postpartum periods are times when women and young families have more intensive contact with healthcare professionals. They are also periods of great change and increased demands on parent’s time, which may result in difficulties establishing or maintaining previous healthy behaviours [14]. Given the closer contact with healthcare services, these times represent a golden opportunity for professionals to support parents in establishing and maintaining healthy behaviours and optimising their family’s wellbeing.

In England, there are five mandated contacts for children aged 0–5 in the universal health visiting service [15]. An NHS trust in the south of England, offers an enhanced health visiting (EHV) programme, an intensive health visiting service for those identified as most in need of support. Families on the programme may have been enrolled because of a range of concerns including poor parental physical or mental health, parental substance abuse, difficulties with child attachment, domestic abuse, previous safeguarding or neglect concerns or difficult social situations such as unemployment or homelessness (amongst other issues). HVs visit up to 31 times in the period from pregnancy until the child is three, usually in person in the parent’s home. Contacts are frequent at the start of the programme and reduce towards the end of the programme. This frequent contact enables HVs to develop relationships with families over time, which makes the HVs ideally placed to discuss issues faced by very vulnerable families and to support recruitment of participants to this study. The strength of these relationships may also impact on the actions that families take in response to information delivered by HVs.

**Fig. 1:**
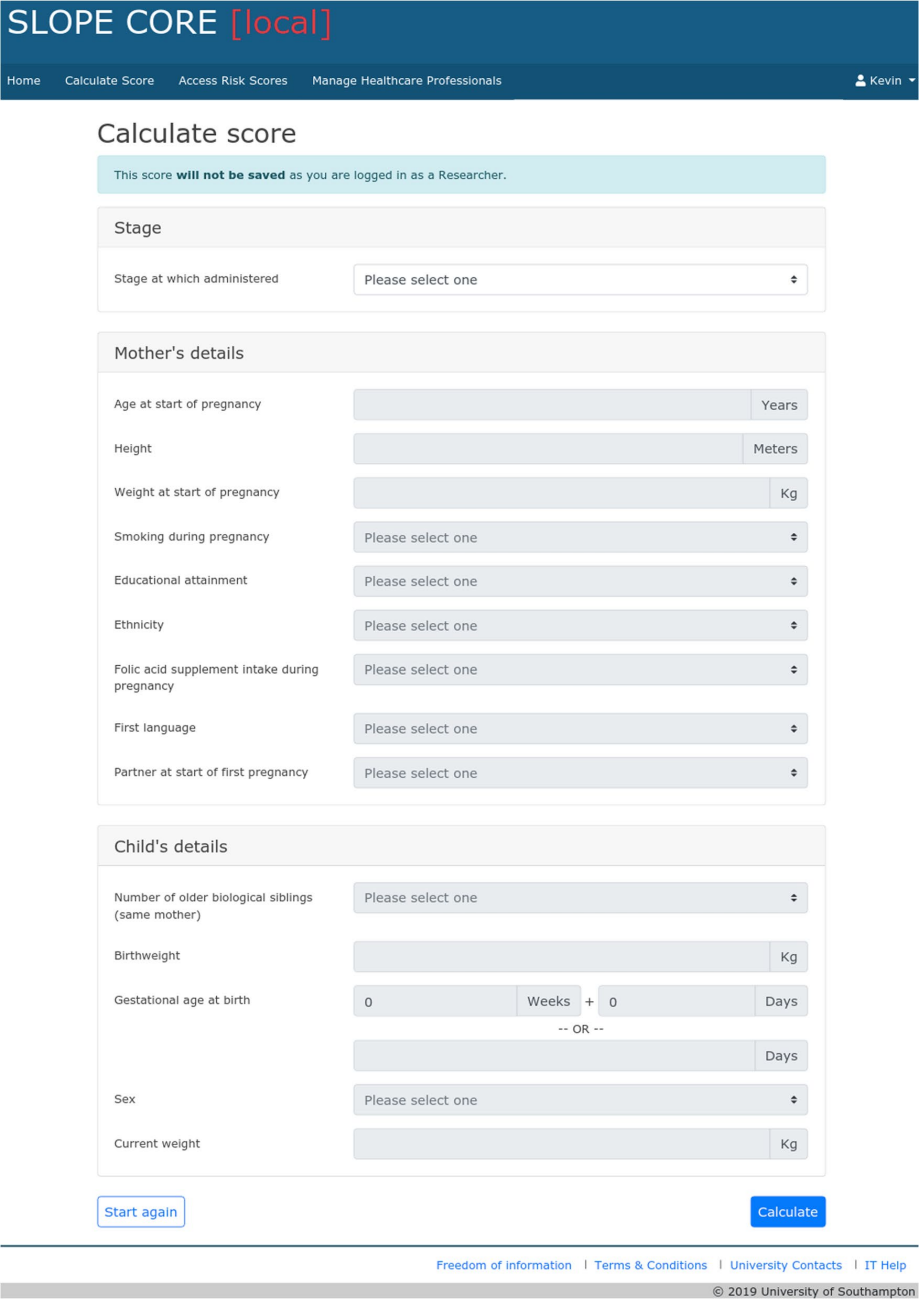
SLOPE CORE online data collection page. This screenshot depicts the data collection page of SLOPE CORE, as viewed through an administrator account for illustration purposes. When using the live tool, the user enters the required data before pressing ‘calculate’ and being taken to a results page. Data required for the tool is routinely collected at healthcare appointments, and should be available in the mother’s clinical records, and the child’s red book

Predicting which children are at high risk of overweight and obesity at an early stage could allow any available support to be targeted towards families at greatest risk. Prediction tools provide the opportunity to take a preventative approach, working towards supporting and maintaining a healthy lifestyle before a child has gained excess weight. Using data from the Studying Lifecourse Obesity PrEdictors (SLOPE), our research group developed a novel Childhood Obesity Risk Estimation tool (SLOPE CORE). SLOPE CORE was generated using prediction modelling of routinely available maternity and early childhood health data in Hampshire, UK, to estimate the risk of childhood obesity at ages 4–5 years [16, 17], and has been externally validated [18]. The tool can be used at four stages: antenatally, after birth, and when the child is approximately 1 year and 2 years old (up until the child is about 2.5 years old). Predictive factors included in the tools calculations at all stages were maternal BMI, smoking status, ethnicity, intake of folic acid supplements and partnership status. Maternal predictive factors included at some, but not all stages of the model, are: maternal age, educational attainment, first language and parity. All of the maternal predictive factors are routinely recorded as part of the antenatal booking appointment, and should be inputted as was true at the time of the booking appointment. Child predictive factors included at some, but not all stages of the model, are: birthweight and gestational age at birth, infant gender, weight at approximately 1 and 2 years. SLOPE CORE has been developed into a web tool that can be used by health professionals (see Fig. 1 for screenshot of data entry page of SLOPE CORE). Whilst other obesity risk prediction tools exist [19–21], SLOPE CORE has the advantage that it has been externally validated, and uses routinely collected data, so it should be possible to use the tool for the vast majority of infants in the UK, without the need for additional data collection.

Many of the families enrolled in the EHV programme are disadvantaged, with high rates of obesity and limited resources to reduce their risk. Limited formal education and low literacy levels are also common. Therefore the EHV group represents an ideal group in which to test the acceptability and feasibility of SLOPE CORE, in order to ensure that the tool is suitable for those with the greatest need.

Although understanding risk of childhood obesity may result in behaviour change for some, it is unlikely that many families will be able to significantly improve their risk of childhood obesity without additional support. Those with limited resources may find this even more challenging [22]. It is vital that healthcare professionals are able to identify those children who are at high risk of obesity, so that limited resources to support families are targeted towards those who need it most. This study addresses several areas of need by aiming to test the feasibility, acceptability and usability of a childhood obesity risk assessment tool within an EHV service, as one context in which this tool could be utilised.

## Methods

### This study aimed to test the acceptability, feasibility and usability of SLOPE CORE

SLOPE CORE was adapted into a web tool that could be accessed by HVs. HVs used the tool together with each parent, either completing the data entry page with input from the parent or supporting the parent to enter the data themselves. Any data that the parent was uncertain about could be extracted from the notes with consent.

Figure 1 contains a screenshot of the data entry page for SLOPE CORE in a test account.

**Fig. 2:**
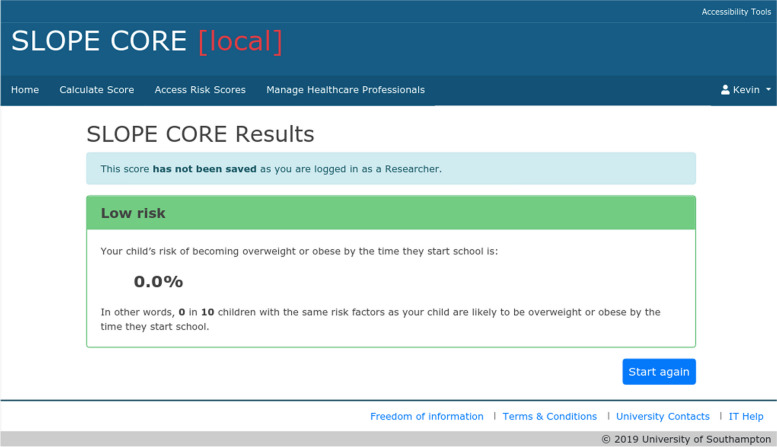
Example of a green risk score results page in SLOPE CORE. The screenshot depicts an example of a low-risk results page (< 20% risk that a child will be obese at age 4–5 years), as viewed through an administrator account for illustration purposes. The risk is conveyed as a percentage and a natural frequency

**Fig. 3:**
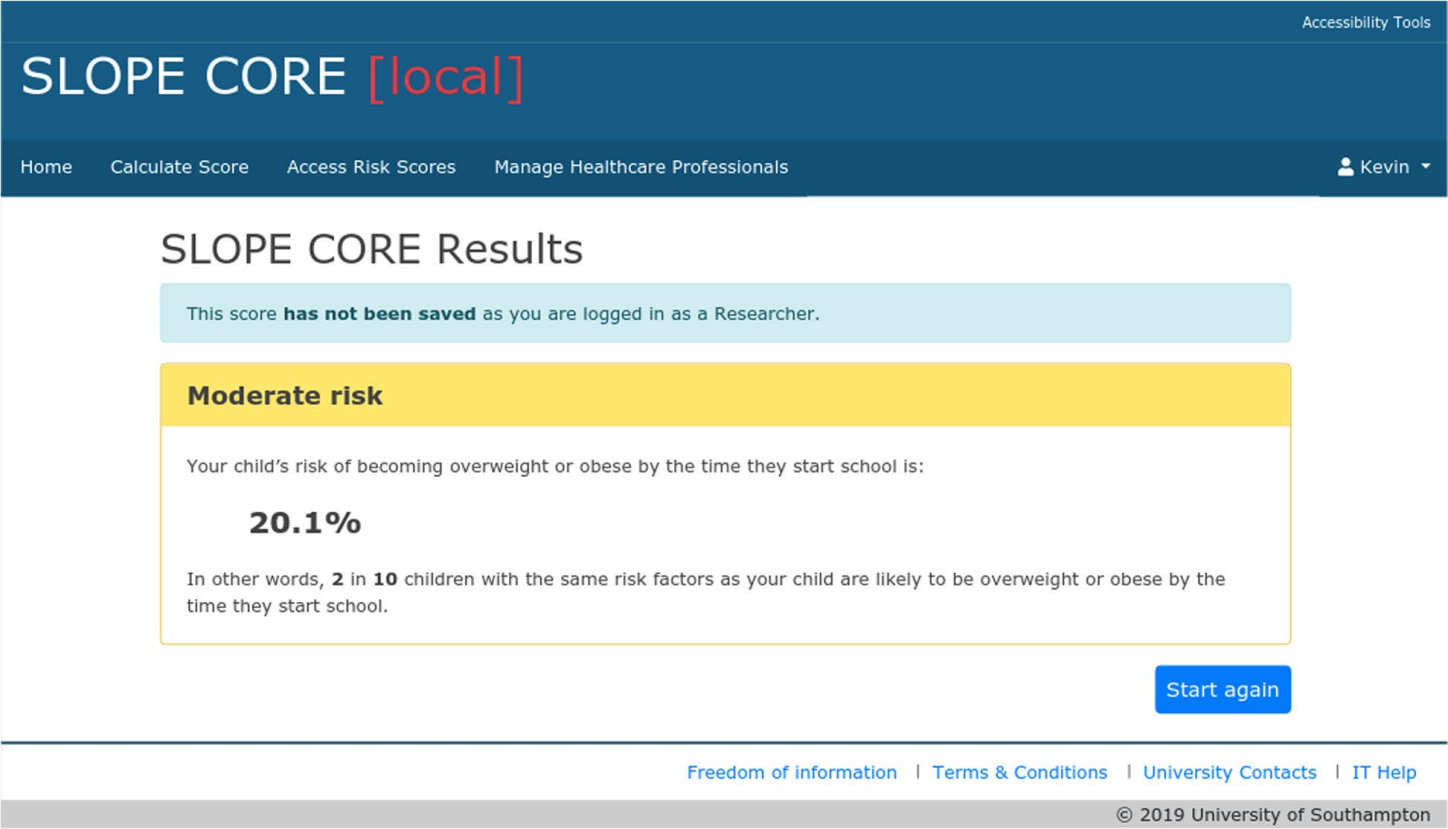
Example of a yellow risk score results page in SLOPE CORE. The screenshot depicts an example of a medium-risk results page (≥ 20 < 30% risk that a child will be obese at age 4–5 years), as viewed through an administrator account for illustration purposes. The risk is conveyed as a percentage and a natural frequency

**Fig. 4:**
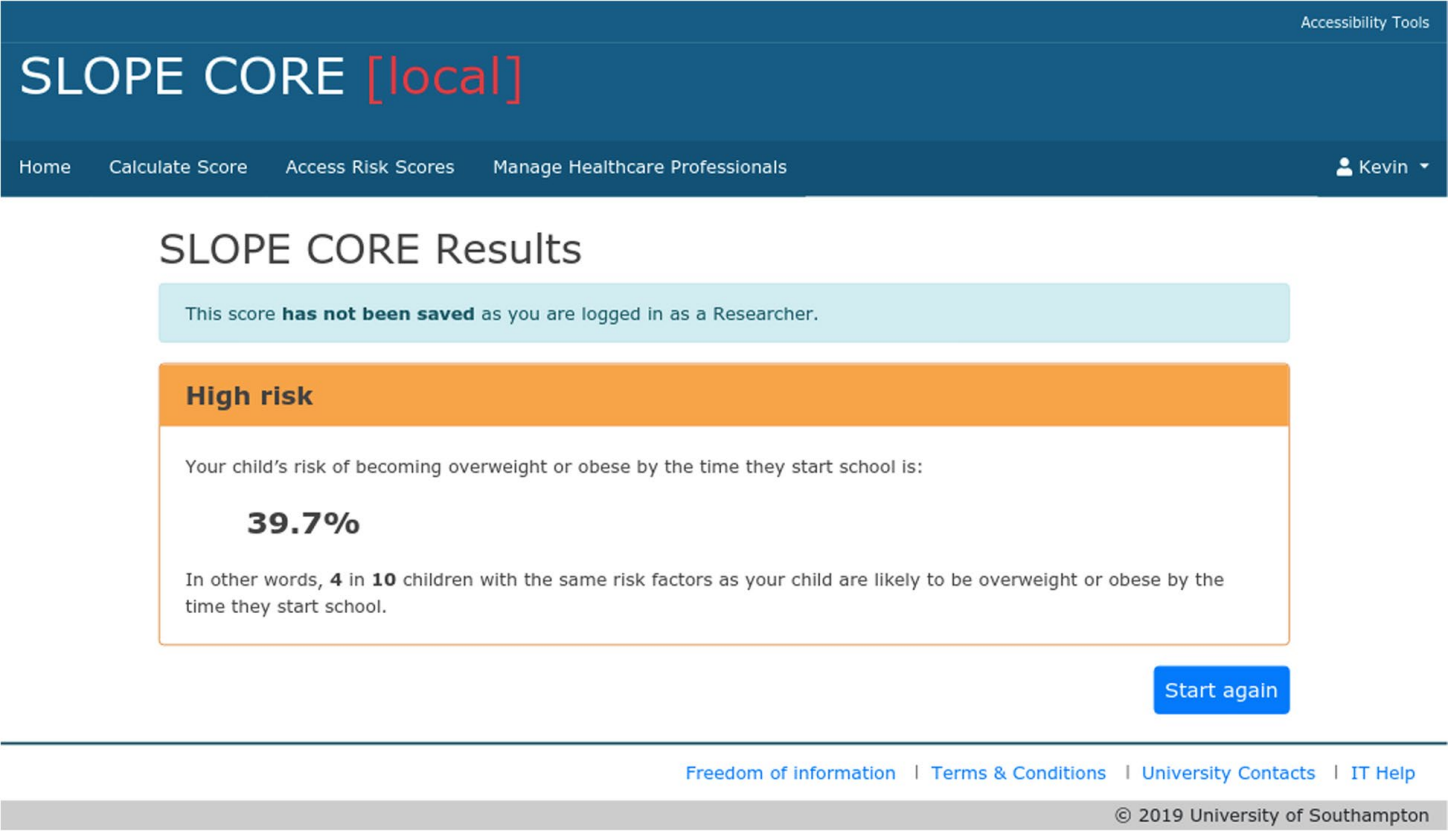
Example of an amber risk score results page in SLOPE CORE. The screenshot depicts an example of a high-risk results page (≥ 30% risk that a child will be obese at age 4–5 years), as viewed through an administrator account for illustration purposes. The risk is conveyed as a percentage and a natural frequency

The results (absolute risk at age 4–5 years) are presented as both a percentage risk and a natural frequency, with a colour coded screen (green for low-risk (< 20%, i.e. there is less than a 20% risk that the child will be obese at age 4–5, Fig. 2), yellow for medium risk (≥ 20 < 30%, Fig. 3) and amber for high-risk (≥ 30%), Fig. 4). There are no clear agreed cut off values that represent high and low risk for childhood obesity, so these ranges were chosen after stakeholder consultation and review of thresholds used by other prediction models. Access to the tool requires a login which can be set up for any users external to the University by the research team. The team considered making the tool openly available but there were concerns about providing resources and managing support for those identified as high risk by the tool, as obesity can be a sensitive topic and thus it was decided to restrict access through the need for a login.

### Study design

This study used a mixed methods approach, using a System Usability Score [23] to test the usability of SLOPE CORE, and qualitative focus groups and interviews.

### Setting and sampling

The study population of HVs was recruited using purposive sampling, from those who were delivering an EHV programme in one NHS trust in the south of England, and who were willing to take part. There were no exclusion criteria for HV participants. Parents were recruited by HVs from the pool of families that were enrolled on the EHV programme under their care, using convenience sampling, although HVs were asked to include parents from a wide range of backgrounds where possible. Parents had been enrolled on the EHV programme for a variable amount of time before being approached about the study, but all had at least met their HV once before. Inclusion criteria for parents were pregnant women or parents with children under 2.5 years of age (as the tool is valid for use from the booking appointment of pregnancy until the child is around 2.5 years old) and being proficient enough in spoken English to enable participation in an interview. Exclusion criteria were those with twins or higher order multiples, children with significant morbidity or following special diets, and children with an existing clinical diagnosis of overweight or obesity. Professional judgement on the part of the HV, along with study inclusion and exclusion criteria, was used to determine eligibility to take part.

### Recruitment and training

HVs were informed about the study by service team leads and through staff meetings attended by the researcher. Those who took part in the study were trained in use of SLOPE CORE, and then asked to use the tool with parents who met inclusion criteria and consented to be a part of the study. Training for HVs comprised one 90 min online session, which took the HVs through an overview of the project and the current weight management pathways, training in study protocols including inclusion and exclusion criteria, participant (parent) recruitment, consent and use of the tool. Training also covered the next steps in the research process for the HVs, such as completing the usability questionnaire and being invited to a focus group. Participants had the opportunity to practice using the tool with a test log in, and to ask questions of the study team. In total 15–20 min of the training session was dedicated to the tool itself and the remainder of the time spent on study processes and weight management pathways.

Recruitment of parents was undertaken by HVs, due to the relationships that they have developed with the families under their care [19, 20]. HVs informed the parents and provided written information about the study initially. In a subsequent contact the HV then obtained consent from participants and used the tool. HVs were not expected to alter their practice based on the tool result, but could follow existing healthy weight pathways if they felt that this was appropriate based on their professional judgement of the child’s growth and weight.

### Data collection and analysis

After using SLOPE CORE at least once in practice, HV participants were invited to attend an online focus group and complete a SUS questionnaire [23]. The SUS consists of 10 questions on a Likert scale (ranging from strongly disagree to strongly agree), which are then scored to give an overall score [23]. Responses which imply a more usable tool attract more points than those that indicate difficulties using the tool. Examples of questions asked include “I found the system unnecessarily complex” and “I found the various functions in this system were well integrated” [23]. The higher the score the more useable the system [23], in general, a score of 70 or above is considered to indicate good usability, whilst a score of below 70 would suggest that further work is needed to improve usability [24]. A review of the SUS found it to be a robust way of rapidly assessing usability [24]. The SUS questionnaire could be completed online by the HV, at a time convenient to them.

The study team also undertook focus groups with HVs and interviews with parents between March and May 2021, to explore experiences of using the tool. Focus groups were chosen for the HV participants in order to generate group discussion, but, after consultation with Patient and Public Involvement (PPI) representatives, it was felt that individual interviews would be more appropriate for parent participants, due to the sensitive nature of the topics being discussed. Focus groups and interviews were conducted online, as a result of COVID-19 restrictions. Focus groups for HVs lasted approximately 60–90 min, and individual interviews for parents lasted approximately 20–30 min. This time included the discussion of other topics, not detailed in this paper. Both focus groups and interviews were recorded using Microsoft teams on a password-protected computer and were conducted using the same topic guide, which was created following PPI consultation.

The data was transcribed using MS Teams transcription software, and then checked for accuracy and edited as appropriate to correct errors and inaccuracies by GG. The data were converted into an anonymised format for analysis, and NVivo software was used to facilitate analysis of the data. The data was analysed using thematic analysis, following Braun and Clarke’s thematic analysis framework [25]. Part of the data (approximately 30%) was double coded by JVA and then codes compared, to ensure coding rigour, any discrepancies were discussed and final interpretations agreed. The completed consolidated criteria for reporting qualitative research (COREQ) checklist [26] is available in Supplementary materials.

### Ethics

Ethical approval was obtained through the University of Southampton and the Health Research Authority, Research Ethics Committee (ERGO number 53387.A4, IRAS project ID 274818, REC reference 20/SC/0099). The appropriate local approvals were also granted before study commencement.

### Patient and public involvement work

PPI contributors were consulted both prior to and during the course of this study. Two contributors have worked with our research group in the past and sat on our steering committee. A further four contributors were consulted during the course of the study, after responding to an advert for PPI contributors which was distributed through Sure Start centres, PPI mailing lists, Facebook groups, twitter and colleagues to share with their networks and family and friends. Contributors were from a range of ethnic backgrounds (White British, Black and African) and were consulted on key issues, including perception of SLOPE CORE and obesity risk, clear communication of risk results and what additional support could accompany SLOPE CORE.

## Results

In total, four HVs were recruited to the study, and successfully used SLOPE CORE tool with parents on the EHV programme. One further HV who had working knowledge of the tool was recruited to take part in focus groups without using SLOPE CORE, and contributed to the wider discussion. In total, SLOPE CORE was used with seven parents (all female), six of the parents received a low risk score for their child, and one parent received a high risk score for their child. Five of the parents who had used the tool gave consent to be contacted about qualitative work, and three of whom were interviewed.

The demographic characteristics of parent participants who used SLOPE CORE (*n* = 7) indicate that the tool was tested with families from a range of backgrounds, including those in disadvantaged groups. All parent participants were female, with an average age of 27 years, and had between 1 and 4 children each. Six of the participants had college age (16–18 years old) education or lower, five were unemployed and six lived in an area of relative high deprivation. Four participants described themselves as being from a White ethnic background, two from a Black African/Caribbean background and one from a Black European background.

Parent participants who took part in an interview (*n* = 3) had an average age of 27, between 1 and 2 children each, and had college or university level education. Two participants described themselves as being from a White ethnic background and one from a Black African/Caribbean background. Two participants were employed, and one lived in an area of relative affluence (IMD decile 7). Those who took part in interviews had higher education and employment levels than those who chose not to participate.

### System usability score

HVs who had used SLOPE CORE completed a system usability questionnaire, and each of their responses were converted to a system usability scale score. The raw responses can be seen in Table 1. SLOPE CORE scored a mean of 84.4 (range 70–97.5), indicating excellent usability. There were no questions that received negative responses, but there were three that received neutral, rather than positive, responses (see Table 1).

**Table 1:** HV responses to SUS questionnaire

	**Responses (*N* = 4)**				
Question:	Strongly disagree	Disagree	Neutral	Agree	Strongly Agree
**I think that I would like to use this system frequently**	0	0	2	0	2
**I found the system unnecessarily complex**	2	2	0	0	0
**I thought the system was easy to use**	0	0	0	2	2
**I think that I would need the support of a technical person to be able to use this system**	3	1	0	0	0
**I found the various functions in this system were well integrated**	0	0	2	1	1
**I thought there was too much inconsistency in this system**	2	2	0	0	0
**I would imagine that most people would learn to use this system very quickly**	0	0	0	2	2
**I found the system very cumbersome to use**	3	1	0	0	0
**I felt very confident using the system**	0	0	2	0	2
**I needed to learn a lot of things before I could get going with this system**	2	2	0	0	0

Questions for the SUS taken from Brooke J. SUS: A “quick and dirty” usability scale. In: Jordan P, Thomas B,* Weerdmeester* *B, *et al*. (eds.) Usability evaluation in industry. London: Taylor and Francis; 1996 p189-94*

### Qualitative data: summary of main themes

The main themes that emerged from the data were the ease of use and potential barriers to use, the presentation of the tool results, the impact of the tool for parents and for HVs, and the tool providing an opportunity for health promotion and behaviour change.

#### Ease of use, and potential barriers to use of SLOPE CORE

Parents that took part in interviews (*n* = 3) reported that they found the tool quick and easy to use, and were happy with how the tool works in its current format.

*It was quite easy to use to be honest. It was really, it was really good. (Parent 2)*

Parents identified that inconsistent internet access for the HVs device may be a barrier to access for some, potentially solved by logging onto the parent’s home WI-FI, if available. When discussing improvements to the tool, parents suggested that including links to, or further resources and support would be of use.

*[on discussion of a higher risk score]… maybe it should just be clear, this is something that you should now sit down and talk with your health visitor about. I think it should say that regardless [of your score]… if you want help with healthy eating or whatever, you can visit this website. (Parent 1)*

HVs also found the tool to be quick and easy to use in practice, with all the information needed being readily available from the parent’s maternity notes or the parent themselves. Some HVs used the tool together with the parents, or let the parent take the lead in inputting information into the tool.

*I've used it twice now and I found it really, really simple to use…. I'd forgotten how quick it was to use. It was done obviously with the parent there and it was just I just, I just found it really simple and straightforward and useful. (HV1)*

Similarly to parents, HVs raised needing to have an internet enabled electronic device, and access to patient notes for accurate maternal height and weight measurements, as a potential barrier to use of the tool in practice. A larger potential barrier that was identified was the need for time and an appropriate opportunity to raise an issue that may be sensitive, all HVs agreed that it would be more difficult to use the tool with a parent that they hadn’t met before, and some were concerned about spoiling an otherwise ‘happy’ contact (such as a new birth visit) with a difficult conversation that a parent may view as negative. However, HVs recognised that discussing weight and dietary behaviours/physical activity is a key part of their role, and so felt it was just a matter of finding an opportunity to raise the issue sensitively.

*It's not a conversation that can be done in sort of 10 minutes, but you know you're going to leave them uncontained, your gonna leave them going away worrying about it, and that will just, you know, just wouldn't be empowering. (HV1)*

*I think we need to be sensitive. I think it's going to be a, it will be difficult if you're told that your child's at high risk of being obese. It's a really touchy subject. So it's doing it as sensitively as possible, but also showing that you know the risk is real and that change does need to happen, so that's going to be the balance. (HV2)*

HVs suggested two potential time points at which they felt the tool could best be utilised, as part of an antenatal EHV contact or at approximately 3–4 months of age when HVs would be talking about the introduction of solids.

*I think ideally a three to four month contact would be the ideal position, because that's when you're going to be focusing and talking about the weaning that will hopefully be not happening before six months. (HV2)*

*In the EHV programme, we do see mums four times antenatally. So we've got a real window of opportunity there to have the, the point of the EHV programme is to be anticipatory so actually for me it feels that having those conversations, using that tool antenatally, is the best opportunity we know parents are more receptive to advice when they’re antenatal, instead of postnatal. So actually I felt that if I was out using that tool in practice I’d quite like to use it antenatally. (HV4)*

#### Presentation of the tool results

Parents felt that the result of the tool was easy to understand, with some finding percentages easier to understand and other preferring the natural frequency, most appreciated using both to improve understanding of the score. One parent thought that the concept of risk prediction can be confusing, and that this may need to be clearly explained by a healthcare professional when using the tool.

*Maybe explain that it's a, it's a probability, not an absolute prediction? I don't know how that can be done, it just explain this is is, this is, uh, it's probably that it may happen. It's not, it's not absolutely going to happen. (Parent 3)*

HVs found the coloured results page was powerful, although some were concerned that a high-risk result may be off putting for some. Similarly to parents, HVs agreed that having both the percentage and a natural frequency was useful, but acknowledged that some of their parents would not grasp either and would need more explanation from the HV to understand the meaning of the result.

#### Impact of the tool for parents and for HVs, and the tool providing an opportunity for health promotion and behaviour change

In general, parents felt that knowing their child’s risk was a good thing, and felt that a low-risk score would provide reassurance that their child was on the right track, whilst a higher risk score provided an opportunity for both healthcare professionals and themselves to intervene to improve their child’s health.

HVs reported several impacts of the tool for parents, primarily relief if their child was classed as low-risk. One concern from discussion about the tool with stakeholders prior to this study was that a low-risk score might result in parental complacency, and potentially reduced efforts to ensure healthy diets and lifestyle for their child. However, HVs felt that, on the contrary, the likelihood of complacency was low, and a low-risk score provided an excellent opportunity for encouragement, praise and positive reinforcement of healthy behaviours. HVs recognised the need to give the result alongside health promotion messaging encouraging parents to maintain a healthy lifestyle for their family.

One HV reported that one parent had a negative reaction to the tool. The parent felt that the tool would have caused her some distress and concern about her weight, had it been used during pregnancy in a ‘real life’ rather than a research context.

*She felt quite uncomfortable about the weight question about her own weight during pregnancy…. she was quite keen to tell me that she felt that if I had used it with her and pregnancy it would have impacted how she felt about her weight…. she said that if I had done it with her pregnancy, she would have been more restrictive with her eating and really worried about her weight. (HV4)*

HVs were concerned about how parents may view the tool and the questions that they were being asked, particularly that some parents may feel judged. However, some reflected that parents seemed to respond more positively and think less about the tool than they expected, which is reflected in the parents’ views of the tool. All the HVs agreed that, although the tool may not be suitable for some parents, such as those suffering with an eating disorder, or who may find the conversation particularly distressing, for the majority a sensitive approach would mitigate any potential negative impacts from using the tool. Some HVs felt that, for a parent for whom the tool may not be suitable to use in partnership, using the tool away from the parent would still inform and benefit the HVs practice for that family.

*If it does come out red [high-risk], it's about containing them… I mean, you just don't want to leave them worrying about that, you know, someone might not worry and think oh well, I don't believe that [score], he looks fine, but others might then be panicking about that and you’ve gotta be mindful that you know you need to have time to contain them and say look, it's a predictor. It's not. You can change this. You know, we can change this…’ (HV1)*

In general, HVs felt that having the conversation sooner rather than later was a positive, as this left more time to change behaviour, healthy weight maintenance from birth or early infancy is easier to achieve than weight loss in an already overweight, older child. HVs felt that having to break the news of a high-risk score after they had already been working with the family for some time and giving routine feeding advice would be a particularly difficult conversation. They felt that this might lead to confusion and mistrust in the HV, for parents who had followed routine advice but whose child was still high risk.

HVs also discussed several impacts of the tool on their own practice. HVs agreed that the tool had several advantages including providing an opportunity for health promotion, encouraging HVs to raise the issue of weight, diet and lifestyle, helping to tailor service delivery, influencing the HVs practice by increasing the amount of anticipatory guidance given to families at high risk, and saving time for those families who need the healthy weight pathway. Possibly the most positive benefit was providing ‘clinical’ evidence (as opposed to the HVs professional judgement) for a child being at high risk. It was felt that this made a difficult conversation easier, by removing personal judgement and focussing on the tool result rather than the HV’s professional opinion. HVs felt that this would be of benefit to their working relationship with the parent and may encourage HVs who are reluctant to have a difficult conversation about weight to broach the issue with more confidence.

*I think it would help us do what we're already doing, but be more clear and be able to be more targeted towards those families that are at higher risk…. (HV4)*

*I think it is easier to talk about it when you have a tool because it's almost the tool giving the answer. It's not like the health visitor is the bad person saying that the child is overweight it’s actually the tool’s giving the answer and the health visitor is there to support you get a better answer in the future. (HV2)*

When considering potential improvements to the tool, most agreed that signposting to additional resources, or a prompt to remind the HV to discuss the next steps, (the next steps being at the discretion of the HV and tailored to the individuals under their care) would be useful. Other potential improvements to the tool mentioned were providing a broader range of ethnic backgrounds to choose from and enabling weights and heights to be entered in either metric or imperial units.

## Discussion

SLOPE CORE provides a quick (a few minutes at most) and easy way to assess a child’s risk of future obesity, using readily available, routinely collected data, which is reliably captured for the vast majority of families. Our small sample of HV participants found the tool easy to use, felt that the tool provided an opportunity for health promotion, and helped to facilitate difficult conversations around weight, diet and lifestyle. The tool was also acceptable to our parent participants, albeit a small number, who wanted to be informed of their child’s risk of obesity, so long as this was accompanied by further information, advice or support. It is important to note that, whilst using the tool itself is very quick, time is needed for an accompanying discussion supporting parents, whatever their result.

### SLOPE CORE

Previous work in obesity risk prediction has suggested that proficiency using technology and ease of use are key barriers to the use of digital risk prediction tools during home visits [20]. HVs felt that SLOPE CORE was easy to use, with a mean SUS score of 84.4 indicating excellent usability. There were no individual scores below the ‘good’ usability threshold (70/100). The SUS is used across a wide range of systems and fields, and has been found to be a reliable measure of usability [24]. A large review of 2,323 different surveys, used over a 10 year period, found a mean SUS score 70.1 (range 0–100) [24], indicating that the usability of SLOPE CORE is better than average. There were some questions with neutral, rather than positive responses. One such response was around confidence using the tool, which might be expected to improve over time. Another, ‘I would like to use this system frequently’ perhaps reflected concerns about the impact of the tool, or having sufficient time to have a sensitive discussion around risk of obesity. Minor changes to the tool suggested by participants could further improve its usability (such as allowing data to be entered in either metric or imperial units). Both parents and HVs reported that needing internet access in order to use the tool could be problematic if the contact took place in the parent’s home, but, if issues were encountered then using a paper version that could be completed later, or using the parent’s home WIFI (if available) could overcome this issue. Concerns were raised around level of understanding of risk in the general population, which is acknowledged to be a challenging concept that is poorly understood by many [27]. Both parents and HVs felt that giving risk scores in percentages and natural frequencies together, went some way to mitigating these concerns, although it was clear that, for some families, SLOPE CORE and its results may need more thorough explanation from the professional administering the tool. Additional training in risk communication for the healthcare professionals using the tool could help to support these conversations, and it’s possible that adding a diagram or pictorial representation of the result may also help to support understanding of risk, as used in other areas of risk communication in healthcare [28, 29].

Risk perception is known to influence behaviour, both in positive and negative directions [10]. It is possible that, if given a low-risk score, some parents may be falsely reassured or complacent, and so deprioritise healthy lifestyles. However, HVs felt that using the tool provided an opportunity for positive reinforcement of good behaviours, and a broader discussion around healthy lifestyle, which may mitigate this concern. Additionally, the parents interviewed expressed a preference for the tool to be accompanied by further discussion with their HV, signposting to further information regardless of risk score, and possibly more support where this was relevant. Perhaps indicating that those parents were interested in improving their lifestyle even if given a low risk score. It is important to note that, although HV and parents discussed the reassuring nature of a low risk score, a low risk score for future obesity may be indicative of other health issues, such as failure to thrive or feeding difficulties etc. Healthcare professionals administering the tool would need to consider the individual in a holistic manner, taking into account their history and the tool’s result before deciding what further advice or support was needed.

When considering using the tool in practice, HVs were concerned about the impact of discussing obesity, which they felt was a sensitive subject, on their relationship with the families under their care, leading to a reluctance to broach the issue for some HVs. Many such concerns have been previously reported in the literature [20, 30] and by HVs during the planning stages of this study. HVs were also concerned about the potentially stigmatising nature of some of the questions (predictors) included in the tool, especially given that several of the predictors are non-modifiable. However, as both the HV and parent participants identified themselves, obesity is a key public health issue, and weight needs to be addressed in order to ensure a holistic view of a child’s health is considered. This illustrates some of the tensions that HVs face when working with families under their care, balancing the importance of building and maintaining a good relationship with their clients, whilst at times having to broach difficult subjects [31]. It is clear from both our findings and the broader literature that work must be done to ensure that healthcare professionals feel empowered to have conversations around weight, diet and lifestyle, in a sensitive manner, when appropriate [30]. Interestingly, HVs reported that, in general, parents taking part in this study were happy to use the tool, and their reactions were more positive than expected.

A recent meta-synthesis of qualitative studies by Bradbury et al., looking at discussing a child’s weight with parents, found that opportunities for health promotion are important in allowing healthcare practitioners an opening to discuss weight [30]. HVs agreed that the tool provided an opportunity for health promotion, and that it made it easier to raise the topic and discuss risk, by using the objective ‘clinical’ evidence from the tool as the basis of their discussion, rather than their subjective professional judgement of the child’s risk. Bradbury et al.also report that the use of an objective tool is helpful in facilitating discussions around weight, and reducing concerns around stigmatising families [30]. Finally, HVs felt that using the tool at an early stage would allow for an easier conversation, as the focus was on prevention, rather than judgement of parenting choices made thus far. They felt that this was likely to make parents more receptive to the discussion, a finding highlighted by Bradbury et al. [30]. HVs also noted that there were several timepoints at which a conversation about weight of the child may be better received, for example, during the antenatal period or about 3–4 months of age, before weaning. Although this may not be suitable for all, in both of these time periods parents are likely to be considering how they want to feed their child, and may be more receptive to a conversation about weight, their child’s risk of overweight and obesity and what they can do to reduce that risk.

### Strengths of the tool

The tool is quick and easy to use, and could provide an opportunity for health promotion and behaviour change. The tool may help to support healthcare professionals to raise a sensitive issue, and facilitate what may be a challenging conversation. Given that the tool uses routinely collected data it is possible that it could be integrated into clinical systems, allowing automatic population of pre-existing data into the tool, saving time for the healthcare professional and avoiding the need to ask potentially stigmatising questions.

### Limitations of the tool

The need for time to discuss a sensitive and complex topic is one limitation of the tool. Risk communication is challenging and sensitivity is required both when taking a parent through the tool questions and discussing the results. Another limitation is that the tool cannot identify low risk results that may be indicative of another health issue, such as failure to thrive. It may not be suitable for all parents at all times, and as such, some use of discretion when deciding whether to use the tool or when is an appropriate time may be required on the part of the healthcare professional administering the tool.

### Strengths of the study

Despite small numbers, a strength of this feasibility study was reaching parents in disadvantaged groups or those facing difficult life circumstances. This, allowed the tool to be tested with those who may have most to gain from preventative strategies but who also may face more barriers to engagement with healthcare or interventions. The demographic data from parent participants highlights the socioeconomic disadvantage that many families enrolled on the EHV programme face. Reaching families facing social and economic disadvantage is challenging, but important, as deprivation, social inequality and obesity are linked [4, 5, 32]. Our sample group also included a larger proportion of participants from ethnic minority groups than would be expected if the group had been representative of the UK population nationally [33]. Additionally, this study sought views from both parents and HVs, obtaining rich data from a variety of perspectives, and allowing for triangulation of the data.

### Limitations of the study

Recruitment to this study was challenging. The health visiting service was impacted by the COVID-19 pandemic, resulting in intense service pressures and a reduced workforce, as well as changes to service delivery. This is reflective of the picture for the health visiting service nationally [34, 35]. As expected, the number of HVs and parents recruited was lower than anticipated. In addition, those parents who chose to take part in interviews had a higher education and employment level than those who used the tool but did not take part in an interview. However, HVs reflected on their experiences and conversations with all the parent participants that used the tool (*n* = 7), so this may have, in a small way, mitigated some the impact of this source of bias. All of our participants were mothers, and so our results do not include any views from fathers. This reflects the predominance of mothers as the main point of contact in the health visiting programme. Additionally, most of our parent participants (6/7) received a low-risk score for their child, which may have impacted their view of the tool.

Several other barriers to parents’ recruitment were raised by, and discussed with, the health visiting service leads. The EHV population is, by definition, vulnerable and high need. Many of the families enrolled on the EHV programme are both socially and economically disadvantaged, and face multiple and competing daily life challenges. Low literacy levels and low educational achievement are common. This means that, in this particular group, the somewhat long and complex Participant Information Sheet (PIS) (including mandatory statements on participation rights and data protection), was off putting, even with the HV taking them through the PIS during a contact. HVs also reported that many parents were initially interested in taking part, but didn’t understand why they needed to wait until the next contact before they could consent and take part. This was a requirement of our ethical approval, allowing participants time to fully consider before consenting and taking part in the research which is considered to be good practice [36], but it resulted in a number of interested families being lost, between the initial contact and being consented to take part. Finally, one parent who consented to be contacted about participating in interviews was interested in taking part, but then declined after other, higher priority issues arose, and another was lost to follow up. It is likely that we would have had a broader range of views if more participants had been recruited to the study. In general, HV participants raised similar issues and were in agreement on most things, perhaps not surprisingly given that data was collected using focus groups, and that the HVs were a professional cohort working in the same area. Parents also reported very similar experiences of using the tool, but it may be that those who felt negatively about the tool declined to take part in interviews, possibly resulting in an overly positive narrative.

### Implications for practice

The small sample size of this feasibility study means that the results require cautious interpretation. Additionally, SLOPE CORE will require further testing to establish how it can be best used in practice, and any impact it may have on childhood obesity, as part of a pathway utilising existing or new interventions to reduce childhood obesity. In an ideal world, the tool would be used sensitively, by a healthcare professional who is appropriately trained in risk communication and well placed to then advise families on the resources available to them and the best next steps. Given the intense pressures facing the health and social care system, an attractive alternative may be to build the tool into existing systems- such as healthy weight pathways, routine HV contacts and relevant GP consultations, which could save time and support existing work rather than further adding to workload for healthcare professionals. If internet access is unreliable, then a paper data collection sheet could be used to capture data required to use the tool at another time.

Once an appropriate setting for the tool use has been established, longer term evaluation considering the impact of the tool will be necessary. The evaluation would need to consider the immediate impact of the tool on healthcare professionals and parents, and whether the tool, in combination with a risk reducing intervention, might be an effective approach to obesity prevention.

Considering the implications for research practice, it was clear that our approach to participant recruitment and consent could be better tailored to our target population. In subsequent work, (not reported here) we adapted our approach to consent based on this feedback. We video recorded participant information, to provide non written options and a more digestible format. We also consented at the same time as giving information about the study. These changes resulted in a smoother consent process that was better received overall.

## Conclusions

This study indicates that SLOPE CORE is potentially acceptable and usable for parents and HVs in practice, and may provide benefit in terms of targeting resources, enabling difficult conversations around weight and initiating early support for children at high risk of obesity. Larger scale trials are needed to confirm the preliminary findings of this study, to establish the best context for use of SLOPE CORE and to understand the impact of the tool on the prevention of childhood obesity.

### Supplementary Information

**Additional file 1. **Consolidated criteria for reporting qualitative studies (COREQ): 32-item checklist. This checklist has been developed from: Tong A, Sainsbury P, Craig J. Consolidated criteria for reporting qualitative research (COREQ): a 32-item checklist for interviews and focus groups. International Journal for Quality in Health Care. 2007. Volume 19, Number 6: pp. 349 – 357.

## Data Availability

The anonymised datasets used and/or analysed during the current study may be available from the corresponding authors on reasonable request, pending approval from the relevant ethics committee and research governance structures. SLOPE CORE is available to trial on request, please contact N.A.Alwan@soton.ac.uk.

## Abbreviations

CORE: Childhood Obesity Risk Estimation tool
COREQ: Consolidated criteria for reporting qualitative research
EHV: Enhanced Health Visiting
HS: Healthy Start
HV: Health Visitor
PIS: Participant Information Sheet
PPI: Patient and Public Involvement
SLOPE: Studying Lifecourse Obesity PrEdictors
SUS: System Usability Scale
