# Supplementary material for: Mixed methods feasibility and usability testing of a childhood obesity risk estimation tool
Source: BMC Public Health. 2023 Sep 4;23:1719. doi: 10.1186/s12889-023-16500-2 (PMC10478378; doi:10.1186/s12889-023-16500-2)
Supplement: Supplementary file 1 — Additional file 1. Consolidated criteria for reporting qualitative studies (COREQ): 32-item checklist. This checklist has been developed from: Tong A, Sainsbury P, Craig J. Consolidated criteria for reporting qualitative research (COREQ): a 32-item checklist for interviews and focus groups. International Journal for Quality in Health Care. 2007. Volume 19, Number 6: pp. 349 – 357. [file 12889_2023_16500_MOESM1_ESM.docx]

Consolidated criteria for reporting qualitative studies (COREQ): 32-item checklist.

Developed from: Tong A, Sainsbury P, Craig J. Consolidated criteria for reporting qualitative research (COREQ): a 32-item checklist for interviews and focus groups. International Journal for Quality in Health Care. 2007. Volume 19, Number 6: pp. 349 – 357

| **Number** | **Item** | **Guide questions/description** |  |
| --- | --- | --- | --- |
| **Domain 1: Research team and reflexivity** | |  |  |
|  | ***Personal Characteristics*** |  |  |
| 1 | Interviewer/facilitator | Which author/s conducted the interview or focus group? | GG, JVA and NAA |
| 2 | Credentials | What were the researcher’s credentials? | GG: BSc (Hons), MBBS, MSc, PgCert HBE, FHEA. MFPH (2019)  JVA: PhD, RGN (at the time, now retired), RHV (at the time, now retired), BA (Hons), MSC, PGCEA, FIHV (at the time, now retired)  NAA: MBE, MBChB, MRCP, FFPH, MPH, MSc, PhD, FHEA, PGCAP |
| 3 | Occupation | What was their occupation at the time of the study? | GG Public Health Registrar, Clinical Academic Fellow and PhD student  NAA: Professor of Public Health  JVA: Professor of Primary and Community Care (now retired) |
| 4 | Gender | Was the researcher male or female? | Female |
| 5 | Experience and training | What experience or training did the researcher have? | GG is a public health doctor with experience of facilitating small groups and had undertaken training in qualitative methods  NAA is a public health researcher with mainly quantitative experience and more recent qualitative training.  JVA is an experienced academic and qualitative researcher. |
|  | ***Relationship with participants*** |  |  |
| 6 | Relationship established | Was a relationship established prior to study commencement? | Researchers had contact with some Health Visitor (HV) participants prior to study commencement, when delivering training to use SLOPE CORE, and when setting up the study. Researchers had no relationship with parent participants prior to the commencement of the study. |
| 7 | Participant knowledge of the interviewer | What did the participants know about the researcher? | Participants knew that GG, JVA and NAA were researchers, and the organisations to which they were affiliated for the purposes of this study. They knew that the researchers were interested in supporting young families to improve their health. |
| 8 | Interviewer characteristics | What characteristics were reported about the interviewer/facilitator? | Authors have all acknowledged their places of work and affiliations. Participants knew that researchers were interested in supporting young families to improve their health. |
| **Domain 2: Study design** | |  |  |
|  | ***Theoretical framework*** |  |  |
| 9 | Methodological orientation and theory | What methodological orientation was stated to underpin the study? | Thematic analysis. |
|  | ***Participant selection*** |  |  |
| 10 | Sampling | How were participants selected? | Purposive (HVs) and convenience (parents) sampling was used. |
| 11 | Method of approach | How were participants approached? | HV participants were approached via email, inviting them to an online information and training session about the study. GG also attended staff virtual staff meetings and an in person training event. Parent participants were approached by their HV, mostly during in person or phone contacts.  HV participants were invited to take part in focus groups via email. Parent participants were invited to take part in interviews via phone and email. |
| 12 | Sample size | How many participants were in the study? | Four HVs used the tool with parents and five HVs attended a focus group. Seven parents used the tool and three completed an interview. |
| 13 | Non-participation | How many people refused to participate or dropped out? Reasons? | Several HVs were interested in taking part but decided not to participate due to work pressures. This research took place in spring of 2021, and the unprecedented service demands of the COVID 19 pandemic impacted both HV and, as a direct result of this, parent recruitment. Of the seven parents who used the tool, two did not give consent to be approached about an interview, one gave consent to be contacted but did not respond to attempts to discuss an interview, and one further parent wanted to take part in an interview but dropped out due to personal circumstances. |
|  | ***Setting*** |  |  |
| 14 | Setting of data collection | Where was the data collected? | Data was collected online via videoconference. |
| 15 | Presence of non-participants | Was anyone else present besides the participants and researchers? | Observers and a research nurse were present for some of the interviews and the focus groups. |
| 16 | Description of sample | What are the important characteristics of the sample? | HV participants were all practicing HVs working for an NHS trust in the south of England. Parent participants were part of an extended health visiting programme, and came from a range of backgrounds. |
|  | ***Data collection*** |  |  |
| 17 | Interview guide | Were questions, prompts, guides provided by the authors? Was it pilot tested? | Interview and focus group topic guides were developed by GG and reviewed by JVA, NAA, DS, NZ and health visiting service leads. Topic guides were not formally piloted, but PPI consultation prior to the study informed the creation of the topic guides. |
| 18 | Repeat interviews | Were repeat interviews carried out? If yes, how many? | No repeat interviews were carried out. |
| 19 | Audio/visual recording | Did the research use audio or visual recording to collect the data? | Yes, audio-visual recordings |
| 20 | Field notes | Were field notes made during and/or after the interview or focus group? | Yes. |
| 21 | Duration | What was the duration of the interviews or focus group? | Interviews lasted for 20-30 minutes, focus groups for 60-90 minutes. |
| 22 | Data saturation | Was data saturation discussed? | Yes |
| 23 | Transcripts returned | Were transcripts returned to participants for comment and/or correction? | No. |
| **Domain 3: Analysis and findings** | |  |  |
|  | ***Data analysis*** |  |  |
| 24 | Number of data coders | How many data coders coded the data? | GG coded the data, JVA double coded some data to discuss emerging themes and check for agreement. |
| 25 | Description of the coding tree | Did authors provide a description of the coding tree? | Yes |
| 26 | Derivation of themes | Were themes identified in advance or derived from the data? | Themes were derived from the data. |
| 27 | Software | What software, if applicable, was used to manage the data? | NVIVO 12 |
| 28 | Participant checking | Did participants provide feedback on the findings? | No |
|  | ***Reporting*** |  |  |
| 29 | Quotations presented | Were participant quotations presented to illustrate the themes / findings? Was each quotation identified? | Yes, quotes are provided in an anonymised format. |
| 30 | Data and findings consistent | Was there consistency between the data presented and the findings? | Yes. |
| 31 | Clarity of major themes | Were major themes clearly presented in the findings? | Yes |
| 32 | Clarity of minor themes | Is there a description of diverse cases or discussion of minor themes? | Yes |
